# Physiological and pathological characteristics of vascular endothelial injury in diabetes and the regulatory mechanism of autophagy

**DOI:** 10.3389/fendo.2023.1191426

**Published:** 2023-06-27

**Authors:** Hanyu Liu, Xueru Wang, Hong Gao, Chan Yang, Chunguang Xie

**Affiliations:** ^1^ Hospital of Chengdu University of Traditional Chinese Medicine, Chengdu, China; ^2^ TCM Regulating Metabolic Diseases Key Laboratory of Sichuan Province, Chengdu, China; ^3^ Division of Endocrinology and Metabolism, State Key Laboratory of Biotherapy, West China Hospital, Sichuan University and Collaborative Innovation Center of Biotherapy, Chengdu, China

**Keywords:** vascular endothelial injury, endothelial cells, pathological characteristics, autophagy, diabetes

## Abstract

Vascular endothelial injury in diabetes mellitus (DM) is the major cause of vascular disease, which is closely related to the occurrence and development of a series of vascular complications and has a serious negative impact on a patient’s health and quality of life. The primary function of normal vascular endothelium is to function as a barrier function. However, in the presence of DM, glucose and lipid metabolism disorders, insulin resistance, inflammatory reactions, oxidative stress, and other factors cause vascular endothelial injury, leading to vascular endothelial lesions from morphology to function. Recently, numerous studies have found that autophagy plays a vital role in regulating the progression of vascular endothelial injury. Therefore, this article compares the morphology and function of normal and diabetic vascular endothelium and focuses on the current regulatory mechanisms and the important role of autophagy in diabetic vascular endothelial injury caused by different signal pathways. We aim to provide some references for future research on the mechanism of vascular endothelial injury in DM, investigate autophagy’s protective or injurious effect, and study potential drugs using autophagy as a target.

## 1 Introduction

Diabetes mellitus (DM) is a chronic metabolic condition characterized by elevated blood glucose levels due to insufficient insulin secretion and/or insulin resistance (IR). According to the International Diabetes Federation research and predictions, the global prevalence of DM was approximately 9.3% (463 million people) in 2019 and is expected to rise to 10.2% (578 million people) and 10.9% (700 million people) by 2030 and 2045, respectively (1). DM has become a killer, endangering people’s lives and health worldwide. The dangers of DM are mainly reflected in its ability to cause extensive vascular complications (2, 3). For example, the risk of cardiovascular disease in patients with type 2 DM (T2DM) is twice that of the general population, and the prognosis is poor (4). About one-third of diabetic patients develop diabetic retinopathy that has a serious negative impact on the patient’s health and life (5). These complications are the most common cause of patient mortality and disability. Therefore, actively exploring the pathological mechanisms of DM and its complications will help develop beneficial preventive measures that can delay and reverse the cacoethic consequences of DM.

As the innermost barrier for tissue to isolate blood, the vascular endothelium can protect tissue from various physical and chemical stimuli to maintain blood vessel function. However, in the presence of DM, the structure and function of the vascular endothelium change, resulting in abnormal vascular barrier function, activation of proinflammatory and procoagulant linkages, increased production of reactive oxygen species (ROS), and decreased bioavailability of nitric oxide (NO), thereby becoming the basis of macrovascular and microvascular damage, which is the initial link in the occurrence and development of diabetic vascular disease (6–8). DM is a complex disease that is accompanied by glucose and lipid metabolism disorders, IR, inflammatory reaction initiation, and oxidative stress activation. Activating autophagy in aortic endothelial cells (ECs) can reduce vascular inflammation and atherosclerosis onset (9) and accelerate endothelial regeneration after injury in DM rats (10), indicating that autophagy regulates vascular endothelium to participate in the progression of vascular lesions in DM. Contrastingly, another mechanism to improve hyperglycemia-induced endothelial injury is achieved by downregulating autophagy (11). In addition, Martino E et al. found that IR and activation of the inflammatory response can induce EC autophagy (12, 13), and autophagy appears to be triggered with the progression of DM. Therefore, it is unclear whether the activation or inactivation of the autophagy switch can inhibit or promote the process of vascular endothelial injury in DM. Therefore, this study aims to evaluate the mechanism by which autophagy regulates vascular endothelial injury in DM and to provide suggestions for the prevention and treatment of diabetic vascular lesions.

## 2 Summary of the physiological structure and function of the vascular endothelium

The vascular endothelium is the primary barrier that protects tissues from circulatory invasion. In terms of morphology, the basic structure of the vascular endothelium is ECs lining the basement membrane. ECs are sparsely distributed along the inner wall of the vessel, and the lumen surface is covered with an endothelial glycocalyx (EG) (14–16). The primary function of the vascular endothelium is to act as a barrier, which not only prevents blood infiltration and maintains blood flow patency but also alters vascular permeability by regulating intercellular connections to allow material transport (17, 18). It also participates in the immune response of inflammatory tissue by regulating blood flow, recruiting leukocytes, and regulating inflammatory factors (14). Finally, ECs can secrete bioactive molecules that regulate vascular homeostasis, such as NO, which promotes vasodilation and cell growth (19, 20); prostacyclin, which promotes vasodilation and anti-thrombosis (21); and endothelin-1 (ET-1) and thromboxane A2, which contract vessels (22, 23). However, the differences in tissue site and vascular type determine the complexity of the structure and function of the vascular endothelium. This article does not focus on endothelial physiology, so it is briefly summarized.

## 3 Pathological characteristics of vascular endothelial injury in DM

The definition of vascular endothelial injury is currently unclear. In the presence of DM, various factors, such as glucose and lipid metabolism disorders, IR, inflammatory reactions, oxidative stress, mitochondrial damage, and activation of advanced glycation end products (AGEs) and their receptors (RAGEs), are responsible for vascular endothelial injury. The major characteristics of vascular endothelial injury include histomorphological changes and dysfunction (24–28), and a systematic description of these characteristics will aid in the development of follow-up studies.

### 3.1 Morphological characteristics of the vascular endothelium in DM

Study done by (29) has shown that the intima-media thickness of the carotid artery increased in adolescents with T2DM and was accompanied by arterial stiffness. In addition, in a DM animal model, the surface of the vascular endothelium was uneven, the ECs were arranged irregularly, most of the ECs were missing and falling off, the remaining ECs contained a large number of cytoplasmic segments and vacuoles, the intima was slightly proliferating or missing, the elastic layer was thin, the boundary was obscure and disordered, and the number of nucleated cells was increased (30–32). This indicates that DM significantly changes the structure and composition of the vascular endothelium. Furthermore, high glucose levels promote ROS production by altering mitochondrial ultrastructure (such as mitochondrial fission and fusion), which is an important feature of endothelial damage (33–35). Moreover, the EG acts as a natural dynamic barrier attaching to the EC surface, and high circulating glucose levels reduce EG’s thickness or eliminate EG (36–38). The loss of EG promotes the adhesion of lipids, monocytes, and platelets to the vascular endothelium, which damages the endothelial barrier and increases endothelial permeability and blood component leakage (39). Importantly, Yan Qiu proved that restoring the thickness and coverage of EG can reduce microvascular permeability and alleviate diastolic dysfunction (40). Thus, reversing EG damage may be the first step in treating diabetic vascular endothelial injury.

Finally, many studies have proven that patients with T2DM have higher levels of circulating ECs than healthy people. This is because ECs exposed to high glucose levels undergo apoptosis (41, 42). High glucose levels inhibit the phosphorylation of AMP-activated protein kinase (AMPK), resulting in the gradual fragmentation of mitochondria. When mitochondrial fusion/division homeostasis is disrupted, ECs can quickly induce apoptosis and vascular injury (43). On the contrary, activating AMPK can inhibit apoptosis induced by high glucose levels and improve endothelial dysfunction (44). Therefore, apoptosis has emerged as an important manifestation of structural and morphological changes in vascular endothelial injury in DM, and it is the main cause of endothelial dysfunction.

### 3.2 Functional characteristics of the vascular endothelium in DM

In the presence of high circulating glucose levels, endothelial dysfunction is characterized by an imbalance of vasodilation and contraction function, an increase in the endothelial permeability and oxidative stress, the release of proinflammatory and prothrombus factors, and leukocyte adhesion (23, 45–47). Importantly, NO produced by L-arginine and nicotinamide adenine dinucleotide phosphate under the action of endothelial NO synthase (eNOS) is not only essential for regulating endothelium-dependent vasodilation but also for mediating various EC functions (48, 49). Therefore, a reduction in NO production or insufficient bioavailability is considered a landmark event in vascular endothelial dysfunction (50, 51).

Some studies have shown that vasodilation inhibition caused by a decrease in NO production or insufficient bioavailability is common in DM (52). In general, glucose can participate in glycolysis via the pentose phosphate pathway, but high glucose levels can reduce endothelial NO bioavailability by inhibiting this pathway (53). The production of AGEs and their interaction with RAGEs can enhance EC permeability (54), inhibit eNOS activity (55), and ultimately prevent NO synthesis. Furthermore, the development of oxidative stress contributes to the reduction of NO bioavailability. High intracellular glucose levels can activate protein kinase C (PKC), which can enhance the oxidative stress response by rapidly inactivating NO, impairing diastolic function, and promoting ET-1 synthesis, thereby resulting in vasoconstriction and platelet aggregation (56). In addition, high glucose levels promote the expression of mitochondrial fission-related proteins, such as dynamin-related protein 1 (Drp1) and fission 1 (Fis1), thereby impairing mitochondrial function, promoting ROS accumulation, reducing eNOS activity and NO bioavailability, and aggravating endothelial dysfunction (57, 58). D’Onofrio N et al. inhibit the oxidative stress response, exert cardiovascular protective effects, and regulate the redox status of endothelial mitochondria by regulating mitochondrial sirtuins (such as SIRT1, SIRT3, and SIRT6) (13, 34, 59). Insulin stabilizes cardiovascular function by stimulating ECs to secrete NO, but IR inhibits the signal pathway of phosphoinositide 3-kinase (PI3K)/v-Akt murine thymoma viral oncogene homolog (Akt) that produces NO, resulting in eNOS inactivation and insufficient NO production (60, 61). The mitogen-activated protein kinase (MAPK) pathway, which produces intercellular adhesion molecule-1, vascular cellular adhesion molecule-1, and E-selectin, is unaffected (25), and cyclic hyperinsulinemia overactivates the MAPK pathway to promote ET-1 (62), ultimately leading to vascular endothelial dysfunction, enhancing endothelial adhesion, and promoting the occurrence and development of vascular endothelial dysfunction.

**Figure 1:**
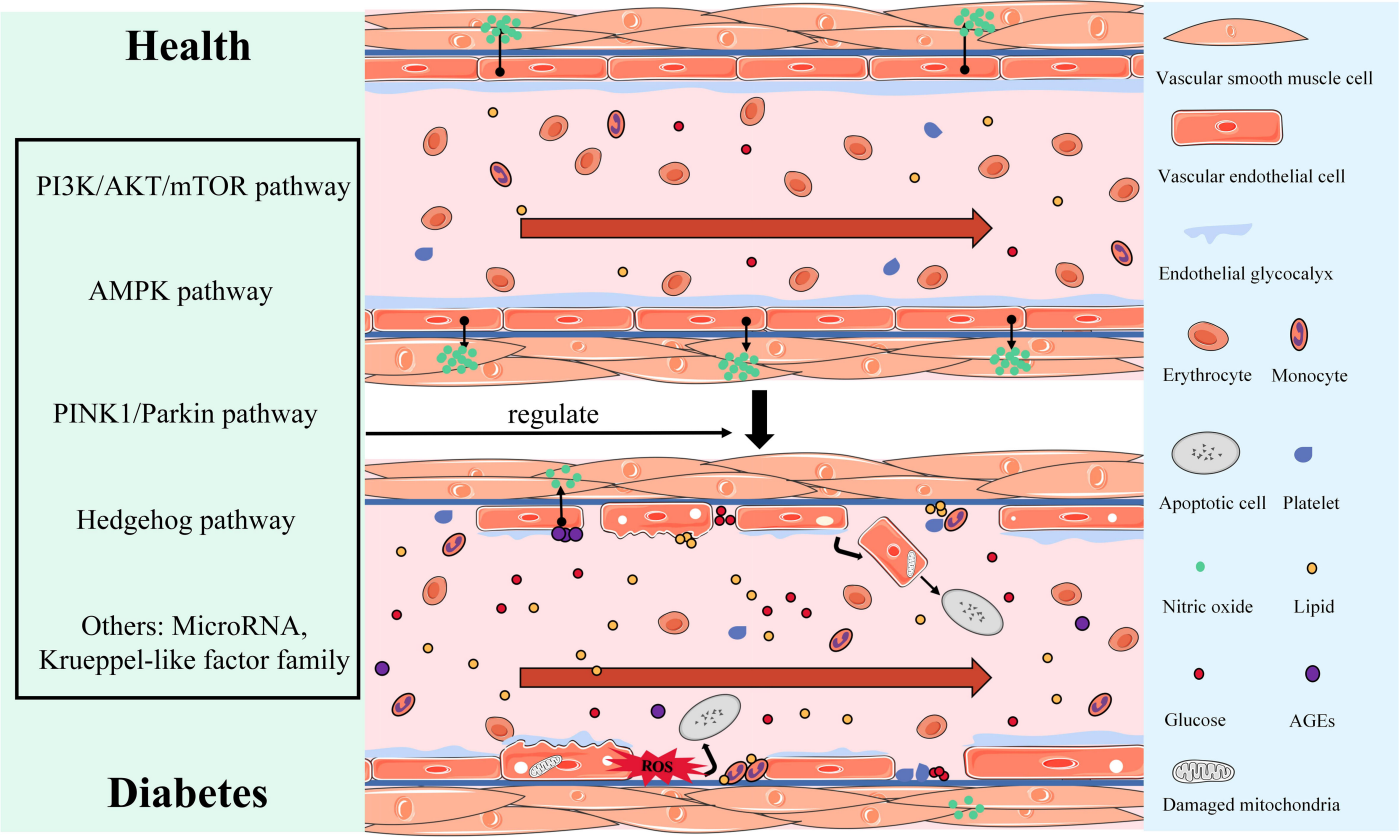
Display of morphological and structural abnormalities and the dysfunction of vascular endothelial injury in DM.

To summarize, the endothelial structure abnormalities, which include morphological changes in the vascular endothelium, mitochondrial damage, ROS accumulation, EG loss, EC apoptosis, and the imbalance of the endothelial dysfunction of vasodilator and vasoconstrictor secretion due to the reduction in NO synthesis or bioavailability, constitute the key pathological characteristics of vascular endothelial injury in DM. **Figure 1** shows vascular endothelial injury.

## 4 Molecular mechanisms of autophagy

For cell homeostasis and body metabolism regulation, autophagy transports intracellular components to lysosomes for degradation and recovery. Autophagy disorder causes the body’s metabolism to shift toward uncontrollable consequences such as IR, DM, and atherosclerosis (63). Autophagy regulation may become an important strategy for preventing the development of cacoethic body metabolism. Mammalian autophagy is classified into three types, namely, macroautophagy, microautophagy, and chaperone-mediated autophagy (64), with megaautophagy being prominent and widely studied. Therefore, this article will focus on introducing the molecular mechanism of megaautophagy (hereinafter referred to as “autophagy”) and its role in vascular endothelial injury in DM.

**Figure 2:**
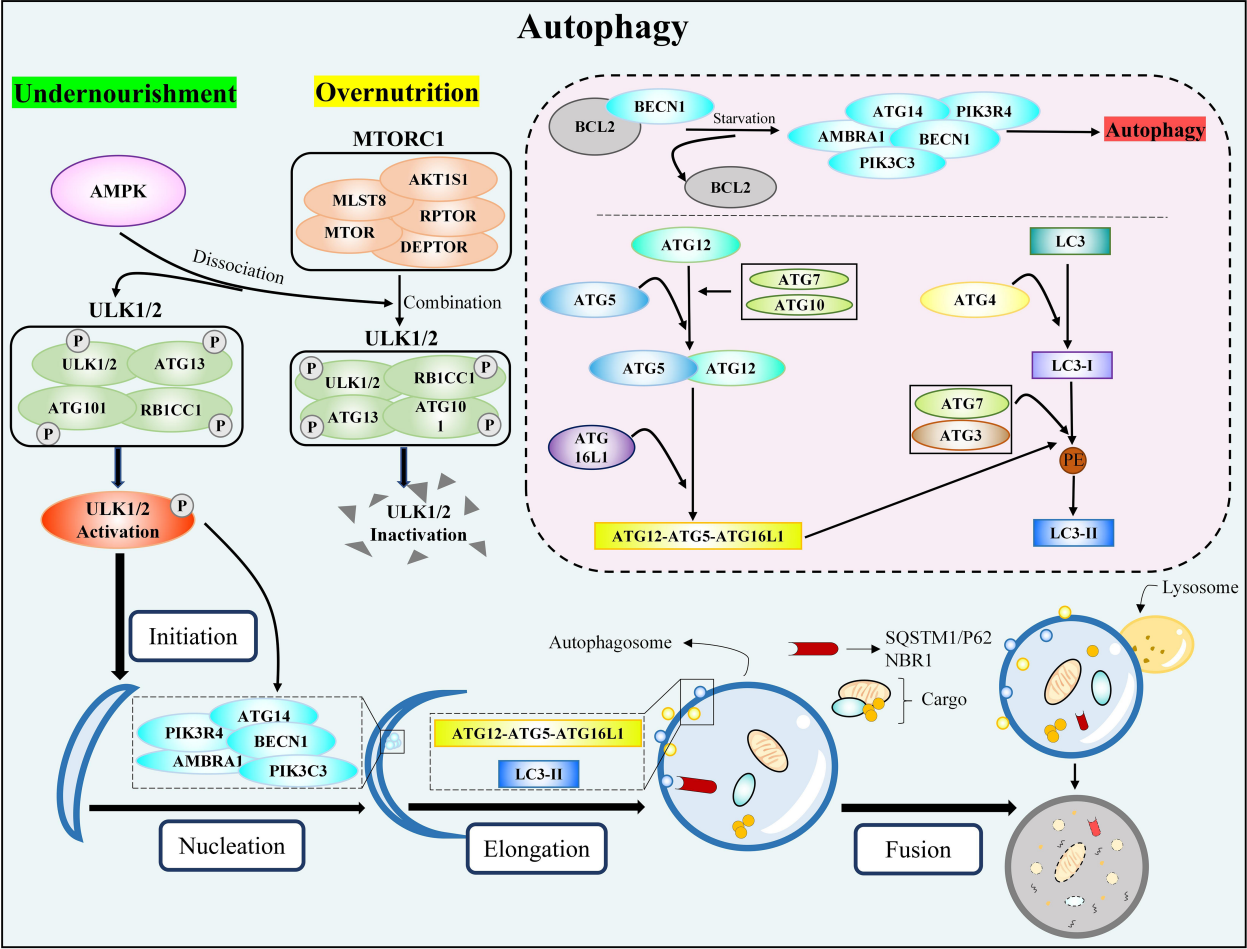
The classic autophagy activation pathway. MTORC1, rapamycin complex 1; ULK1/2, unc-51-like kinase 1/2; ATG, autophagy-related genes; RB1CC1, RB1-inducible coiled-coil 1; AMPK, AMP-activated protein kinase; BECN1, beclin 1; PIK3C3, phosphatidylinositol 3-kinase catalytic subunit type 3; PIK3R4, phosphoinositide 3-kinase regulatory subunit 4; AMBRA1, activating molecule in Beclin1-regulated autophagy protein 1; BCL2, B-cell lymphoma 2; ATG16L1, autophagy-related gene 16-like 1; LC3, microtubule-associated protein light chain 3; PE, phosphatidylethanolamine; SQSTM1, sequestosome 1; NBR1, neighbor of BRCA1 gene 1.

Mammalian autophagy activation includes four key steps: initiation, nucleation, elongation, and fusion. When the body is nourished and energized, the MTORC1 and ULK1/2 complexes (ULK1/2-ATG13-RB1CC1-ATG101) work together to inactivate ULK1/2 and ATG13, preventing autophagy. Conversely, insufficient nutrition or energy causes AMPK to activate MTORC1, dissociating MTORC1 and ULK1/2, partially dephosphorylating ULK1/2 and ATG13, and then phosphorylating RB1CC1 and ATG101, which initiates autophagy (65, 66). The activation of the ATG14 complex (ATG14-BECN1-PIK3C3-PIK3R4) by the phosphorylated ULK1/2 complex is the key step in nucleation. During the nucleation process, AMBRA1 can positively regulate the ATG14 complex and autophagy by preventing BCL2 from binding to BECN1 (66). The elongation process mainly involves two protein-coupling events (67, 68): (1) with the help of ATG7 and ATG10, ATG12 is coupled with ATG5, and the coupling compound is combined with ATG16L1 to finally form the ATG12-ATG5-ATG16L1 complex; (2) LC3 forms LC3-I under the effect of ATG4. LC3-I is coupled with PE on the surface of emerging autophages via ATG3 and ATG7 to form LC3-II; this process is also guided by the ATG5-ATG12-ATG16L1 complex. Subsequently, LC3-II is placed on the autophagy membrane and interacts with receptors responsible for recruiting autophagy substrates (such as SQSTM1/P62 and NBR1), causing the isolation membrane to expand and seal, resulting in the formation of autophagosomes (LC3-II/LC3-I reflects the amount of autophagosome formation) (69–71). Finally, the autophagosome and lysosome fuse to form the autolysosome, which degrades autophagy substrates. The specific activation process of autophagy is shown in **Figure 2**.

## 5 Mechanism of autophagy regulating vascular endothelial injury in DM

Several studies have proved that DM patients have impaired autophagy in the vascular endothelium compared with non-DM patients and that impaired autophagy induces and aggravates vascular endothelium morphological abnormalities and dysfunction, including EC apoptosis and exfoliation (72, 73), EG reduction (72), insufficient EC migration and generation (10), increased oxidative stress (74, 75), and blocked eNOS activation (76). The recovery of damaged autophagy can prevent vascular endothelial injury in DM (10, 76). Therefore, autophagy regulation is expected to become an important strategy for delaying and improving DM angiopathy. However, autophagy can cause excessive consumption of intracellular proteins and organelles, promote degradation of anti-apoptosis proteins and cell survival factors (77), result in autophagic death and apoptosis (78, 79), and subsequently induce EC dysfunction. Therefore, in order to provide insights for future basic research and clinical applications, it is urgent to sift through and summarize the relevant literature data and mechanisms to explore the role of autophagy in regulating vascular endothelial injury in DM.

### 5.1 PI3K/AKT/mechanistic target of rapamycin pathway

In diabetic vascular endothelium, autophagy-mediated EC injury is commonly inhibited by activating the PI3K/AKT/mTOR signaling pathway. Long-term AGE stimulation upregulates forkhead box transcription factor O1 (FOXO1) and enhances AKT activity, which decreases silent information regulator SIRT1 deacetylase activity via phosphorylation and inhibits ATG14 expression. The above mechanism impairs autophagosome–lysosome fusion and mediates autophagic apoptosis in ECs (80). Regulating the PI3K/AKT/mTOR signaling pathway to activate autophagy is an effective way to reduce DM-related EC injury. For example, miR-126, miR-199a-3p, and exosome miR-21 upregulate the expression of autophagy-promoting genes, such as ATG5, Beclin1, and LC3, by inhibiting the PI3K/AKT/mTOR signaling pathway; activate EC autophagy; restore autophagy flux; and alleviate endothelial injury induced by glycolipid metabolism disorders (81–84). Furthermore, in diabetic mice, activating promyelocytic leukemia zinc finger protein (PLZF) can upregulate PI3K expression, promote EC autophagy, and protect the vascular endothelium from AGE-induced injury (85).

However, contrary evidence suggests that autophagy induced by the PI3K/AKT/mTOR pathway aggravates endothelial injury. As the key mediator of vascular injury in patients with DM, methylglyoxal, the precursor of AGEs, significantly activates autophagy and inhibits angiogenesis by inhibiting ROS-mediated AKT/mTOR signaling pathways or inhibits phosphorylation of sestrin 1 (SESN1) and SESN2 and mTORC1 activation by activating p53 to promote autophagy activation and induce EC activity decline and injury (86, 87). Moreover, AGEs can induce excessive autophagy via the PI3K/Akt pathway, impair angiogenesis, and lead to angiogenesis disorders (88). Conversely, activation of the PI3K/AKT/mTOR pathway may mitigate endothelial injury caused by disturbances in glucose and lipid metabolism by inhibiting EC autophagy (89, 90). According to the above studies, although the PI3K/AKT/mTOR pathway has been extensively studied in terms of regulating autophagy, high-quality and rigorous literature is still needed to support its protective or destructive effects on EC injury.

### 5.2 AMPK pathway

It is well known that AMPK can counteract the effects of mTOR on autophagy. High glucose levels directly induce endothelial injury by inhibiting autophagy in human umbilical vein endothelial cells (HUVECs) via the AMPK pathway (91). Downregulation of AMPK and PIK3C3 phosphorylation promotes mTOR phosphorylation, inhibits autophagy activation, and leads to insufficient autophagy degradation of caveolin-1 (CAV1) via CAV1-CAVIN1-LC3-II (caveolae associated protein 1, CAVIN1). CAV1 accumulation in the cytoplasm promotes the transport of low-density lipoprotein (LDL) in ECs and induces EC apoptosis, which serves as the basis for endothelial injury and dysfunction (92–94). These studies confirm that activating the AMPK pathway to reverse EC autophagy inhibition is the key to improving the endothelial injury induced by DM. By promoting AMPK phosphorylation and inhibiting mTOR expression, angiotensin-converting enzyme 2 promotes autophagy, increases plasma NO levels, improves endothelial relaxation function, and corrects endothelial dysfunction (95, 96). In addition, lysosomal damage mediates the activation of the inflammasome-NLR family pyrin domain containing 3 (NLRP3) and the release of high mobility cassette protein 1, leading to endothelial hyperpermeability. However, activating autophagy via the AMPK pathway can inhibit the assembly and activation of NLRP3, restore endothelial connectivity, and reduce endothelial permeability (97, 98). Finally, Weikel KA et al. found an increase in glycogen synthase kinase 3β (GSK3β) activity in DM patients and animals participating in autophagy inhibition. Inhibiting GSK3β expression can ultimately activate FOXO1 by reducing Akt activity and increasing AMPK activity, thereby promoting autophagosome formation, maintaining EC health, and slowing the progression of vascular diseases (99).

### 5.3 PTEN-induced putative kinase 1/Parkin pathway

Autophagy is classified as selective or non-selective based on the difference between autophagy receptor and substrate binding. In selective autophagy, mitophagy is considered the central mechanism for regulating mitochondrial quality and the key to maintaining cell balance (100). The PINK1/Parkin pathway is a well-known mechanism to regulate mitophagy, which affects the progression of DM and vascular complications. Glucose and lipid metabolism disorders not only induce mitochondrial dysfunction by increasing the expression of the mitochondrial fission-related proteins mitochondrial adaptor Fis1 and Drp1/p-Drp1 (78) but also promote protein kinase Cδ (PKCδ)/Drp1 signal transduction, which can trigger hexokinase II (HK-II) dissociated from mitochondria and downregulate the HK-II/PINK1/Parkin pathway to inhibit mitophagy and aggravate endothelial injury (101). On the contrary, activating the PINK1/Parkin pathway to trigger mitophagy improves mitochondrial dysfunction and protects ECs from high glucose-induced damage (78, 102, 103). Zhang MY et al. discovered that Takeda G protein-coupled receptor 5, a new type of bile acid cell membrane receptor, inhibits mitochondrial fission of retinal microvascular endothelial cells and enhances mitophagy to alleviate EC apoptosis and dysfunction due to high glucose levels by regulating the PKCδ/Drp1-HK2-PINK1/Parkin pathway (104). In addition, research shows that the brain-derived neurotrophic factor/tropomyosin receptor kinase B/hypoxia-inducible factor-1α/BCL2-adenovirus E1B 19 kDa protein-interacting protein 3 (BNIP3) pathway can activate mitophagy, inhibit oxidative stress and apoptosis in BMECs under high glucose conditions, and reverse EC injury (105).

However, it has been reported that the PINK1/Parkin pathway is upregulated and activated in the blood vessels of obese and DM mice (106) and that mitophagy activated by the c-Jun N-terminal kinase/p38 pathway will aggravate endothelial injury (107), which is contradictory to the above effects of activating mitophagy to protect the diabetic endothelial injury. Reducing PINK1 expression and Parkin accumulation in mitochondria, restoring SIRT1 expression, and inhibiting mitophagy can prevent eNOS inactivation and promote NO secretion to protect ECs from apoptosis and oxidative stress induced by high glucose levels as well as alleviate EC injury and dysfunction (108, 109). Based on the findings from the above studies, the true function of mitophagy in vascular endothelial injury in DM needs further exploration.

### 5.4 Hedgehog pathway

Niu C et al. proved via *in vitro* and *in vivo* experiments that high glucose levels can promote abnormal EC structure and dysfunction by inducing EC leakage, reducing angiogenesis, and triggering apoptosis. Throughout the process of high glucose-induced endothelial injury, the autophagy marker LC3 of ECs is significantly accumulated and the activation of the hedgehog pathway is impaired. Importantly, activating the hedgehog glioma-associated oncogene homolod-1 pathway and downregulating BNIP3 expression inhibit the binding of BNIP3 and BCL2, enhance the binding of BECN1 and BCL2, and ultimately inhibit autophagy and improve EC injury and dysfunction (11), indicating that autophagy may be an important risk factor for inducing endothelial injury and that inhibition of autophagy will play a role in inhibiting hyperglycemia-induced endothelial injury. The role of the hedgehog pathway and autophagy in regulating the progression of human disease has been discussed in many articles (110, 111), but they are still in the early phases of research in regulating the mechanism of vascular endothelial injury in DM. In the future, we can focus on the cross-interference and therapeutic potential among the three.

### 5.5 Other pathways

MicroRNA is a type of small non-coding RNA that regulates cell growth, differentiation, development, and apoptosis and actively participates in the onset of many diseases, including vascular endothelial injury in DM. Jianbing H et al. reported that the target gene of miR-34a was ATG4B, which was regulated by allograft inflammatory factor-1 (AIF-1). High glucose levels increased AIF-1 expression in human glomerular endothelial cells (HRGECs), followed by an increased miR-34a expression, which inhibited ATG4B expression and EC autophagy to induce inflammation and oxidative stress in HRGECs (112). In addition, Sha W et al. found that inhibiting miR-142-3p expression can promote autophagy activation mediated by its target gene sprouty−related EVH1 domain protein 2, which ultimately plays a protective role in ECs (113). The Krueppel-like factor (KLF) family is a class of transcription regulators that controls cell growth, development, and differentiation. Among them, KLF2 is a key regulator of autophagosome–lysosome interactions. Wu H et al. found that increasing KLF2 expression can enhance autophagy while also inhibiting foam cell formation and apoptosis (114). However, low KLF4 expression impairs SIRT6’s enhancement of autophagy, leading to incomplete endothelium (115).

## 6 Conclusion and outlook

**Figure 3:**
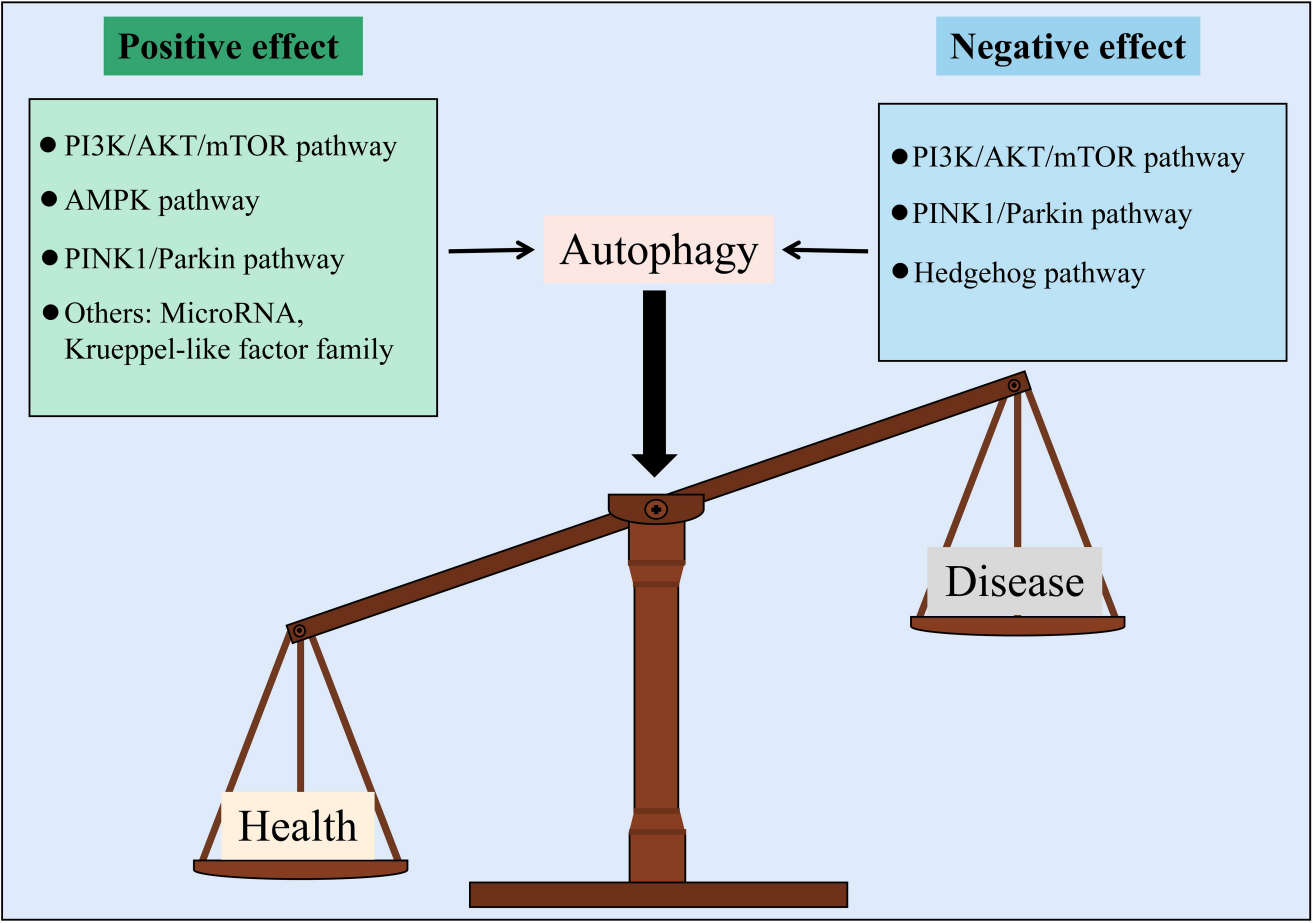
Autophagy is a balance to regulate vascular endothelial injury in DM from two different aspects.

In general, this review summarized the characteristics of endothelial injury in DM and explored the mechanism of autophagy regulating diabetic EC injury. In the presence of DM, the endothelial injury was found to be characterized by ECs with abnormal morphology, mitochondrial damage, ROS accumulation, EG defects, EC apoptosis, and endothelial dysfunction due to a decrease in NO utilization. Autophagy regulates the progression of diabetic endothelial injury via pathways such as PI3K/AKT/mTOR, AMPK, PINK1/Parkin, and Hedgehog, and its regulatory potential is undeniable. However, autophagy is a double-edged sword with positive and negative two-way regulatory effects on diabetic endothelial injury. The ability to control the “degree” of autophagy’s two-way regulation is critical for studying its role in the progression of diabetic endothelial injury (**Figure 3**).

Based on the above mechanism overview, different pathways regulate a variety of downstream targets or are regulated by various upstream targets, resulting in distinct disease progression, which may cause autophagy to play opposing roles in regulating diabetic endothelial injury. First, different signaling pathways have different effects. For example, activating the AMPK pathway can reverse EC autophagy inhibition, which can improve endothelial injury, but the Hedgehog pathway has the opposite effect. Second, in the same signal pathway, the difference in upstream signal molecules leads to the activation or inhibition of the pathway, which plays distinct roles in the end events. For example, miR-126, miR-199a-3p, and miR-21 inhibit the PI3K/AKT/mTOR pathway to activate EC autophagy and alleviate endothelial injury in DM. However, LDL activates PI3K/AKT/mTOR signal transduction to inhibit EC autophagy and alleviate endothelial injury in DM. Even the same upstream molecules might play different roles. For example, the activation of FOXO1 has the opposite effect on mediating autophagy formation and function. Finally, because this paper involves various ECs, such as HUVECs and HRGECs, the effect of autophagy on diabetic endothelial injury is affected by differences in research programs. Importantly, because DM is a complex disease with multiple coexisting and interacting factors, autophagy can play different roles in endothelial dysfunction triggered by different stimuli. Most studies suggest that autophagy activation can protect against vascular endothelial injury induced by high glucose levels (116, 117), but Liu Y et al. found that glucose and lipid toxicities can initiate autophagy and inhibit autophagic blood flow, thereby causing autophagy volume aggregation, inducing apoptosis, and inhibiting EC function (78). As mentioned above, LDL transport through ECs is the foundation of endothelial injury and dysfunction, and LDL stimulation will promote PI3K/AKT activation and subsequent autophagy inhibition (89). Shi G et al. found that the SIRT1/Beclin-1/autophagy axis can protect against LDL-induced vascular aging (118). Inflammatory activation induces endothelial dysfunction by increasing oxidative stress (119), and enhanced autophagy can alleviate inflammation and apoptosis in atherosclerotic ECs and play a cardiovascular protective role (120). However, in high glucose environments, ROS accumulation in HRGECs inhibits eNOS activity and NO bioavailability, which is related to an increase in autophagy flux (121). The role of autophagy in diabetic vascular endothelial injury is uncertain.

In conclusion, the present research suggests that the regulation of autophagy can improve and reverse diabetic endothelial injury. However, due to the complexity of autophagy’s regulatory mechanism and differences in research programs, the specific role of autophagy in regulating diabetic endothelial injury remains unknown. Therefore, a more rigorous and in-depth research is needed to determine the relationship between autophagy and the progression of diabetic endothelial injury as well as to clarify if there is a degree of autophagy in order to determine whether autophagy is beneficial or harmful. In addition, to determine which pathway plays a positive or negative regulatory role, after determining the specific role, differentially expressed genes need to be investigated and verified. This may help control the “degree” of the two-way regulatory effect of autophagy.

## Author contributions

HL and CY conceived this paper. HL wrote this article. XW collected literatures. CX and HG reviewed the manuscript and proposed modification comments. All authors have contributed to this article and approved the submitted final version.
